# Isocytosine deaminase Vcz as a novel tool for the prodrug cancer therapy

**DOI:** 10.1186/s12885-019-5409-7

**Published:** 2019-03-04

**Authors:** Arunas Kazlauskas, Adas Darinskas, Rolandas Meškys, Arimantas Tamašauskas, Jaunius Urbonavičius

**Affiliations:** 1 Laboratory of Molecular Neurooncology, Neuroscience Institute, Medical Academy, Lithuanian University of Health Sciences, Eiveniu str. 4, LT-50161 Kaunas, Lithuania; 2 Laboratory of Immunology, National Cancer Institute, Santariskiu Str. 1, LT-08660 Vilnius, Lithuania; 3 Department of Molecular Microbiology and Biotechnology, Institute of Biochemistry, Life Sciences Center, Vilnius University, Sauletekio al.7, LT-10222 Vilnius, Lithuania; 4 Department of Chemistry and Bioengineering, Vilnius Gediminas Technical University, Sauletekio al.11, LT-10221 Vilnius, Lithuania

**Keywords:** 5-fluoroisocytosine, Isocytosine deaminase, 5-fluorouracil, Prodrug-activation system, Cancer therapy

## Abstract

**Background:**

The cytosine deaminase (CD)/5-fluorocytosine (5-FC) system is among the best explored enzyme/prodrug systems in the field of the suicide gene therapy. Recently, by the screening of the environmental metagenomic libraries we identified a novel isocytosine deaminase (ICD), termed Vcz, which is able of specifically converting a prodrug 5-fluoroisocytosine (5-FIC) into toxic drug 5-fluorouracil (5-FU). The aim of this study is to test the applicability of the ICD Vcz / 5-FIC pair as a potential suicide gene therapy tool.

**Methods:**

Vcz-expressing human glioblastoma U87 and epithelial colorectal adenocarcinoma Caco-2 cells were treated with 5-FIC, and the Vcz-mediated cytotoxicity was evaluated by performing an MTT assay. In order to examine anti-tumor effects of the Vcz/5-FIC system in vivo, murine bone marrow-derived mesenchymal stem cells (MSC) were transduced with the Vcz-coding lentivirus and co-injected with 5-FIC or control reagents into subcutaneous GL261 tumors evoked in C57/BL6 mice.

**Results:**

5-FIC alone showed no significant toxic effects on U87 and Caco-2 cells at 100 μM concentration, whereas the number of cells of both cell lines that express Vcz cytosine deaminase gene decreased by approximately 60% in the presence of 5-FIC. The cytotoxic effects on cells were also induced by media collected from Vcz-expressing cells pre-treated with 5-FIC. The co-injection of the Vcz-transduced mesenchymal stem cells and 5-FIC have been shown to augment tumor necrosis and increase longevity of tumorized mice by 50% in comparison with control group animals.

**Conclusions:**

We have confirmed that the novel ICD Vcz together with the non-toxic prodrug 5-FIC has a potential of being a new enzyme/prodrug system for suicide gene therapy.

**Electronic supplementary material:**

The online version of this article (10.1186/s12885-019-5409-7) contains supplementary material, which is available to authorized users.

## Background

The suicide gene therapy is one of the types of gene therapies designed for cancer treatment. At the core of this therapy is the use of genes encoding enzymes that are able convert a biologically inactive compound (prodrug) into a cytotoxic molecule (drug). The cytosine deaminase (CD)/5-fluorocytosine (5-FC) system is among the best explored enzyme/prodrug systems in the field of cancer therapy [1–5]. The CD deaminates the low-toxic pyrimidine 5-FC, which is commonly used to control the fungal infections [6], into 5-fluorouracil (5-FU), which is further converted to antimetabolites, such as 5-fluoro-2′-deoxyuridine-5′-monophosphate or 5-fluorouridine-5′-triphosphate, sensitizing cells to the lethal effects of radiation therapy [5, 7–9]. Both in vitro and in vivo studies demonstrated that the 5-FU produced upon the action of the CD/5-FC system induce the growth inhibition and apoptosis-mediated cell death of a variety of types of tumor including glioma, mammary, colorectal and prostate cancers [1, 10–15]. The CD/5-FC system is known for its strong “bystander effect” on tumors, which could be due to non-facilitated diffusion of small uncharged 5-FU molecules through cellular membranes. Both 5-FC and 5-FU can penetrate well into most body locations including cerebrospinal, vitreous and peritoneal fluids [6, 16]. The major disadvantage of the bystander effect is the non-specific targeting of normal cells that are located in the vicinity of the tumor [15]. Therefore, the targeted delivery of 5-FU-producing system such as CD/5-FC to the tumor location remains one of the main problems the suicide gene therapy has to face. Among the most promising approaches to solve this problem is the use of adenoviruses [17, 18] or tumor-tropic cells, such as mesenchymal stem/stromal cells (MSC) [19, 20] or neural stem cells [21], as carriers of the enzyme / prodrug system to tumor sites.

There is yet another problem, which persists with the CD/5-FC system, is that 5-FC is not an entirely harmless molecule as would be ideally expected for the cancer therapy. The deleterious effects of 5-FC to the organism ranging from minor side effects such as nausea, vomiting and diarrhea to serious ill-effects such as hepatotoxicity and bone-marrow depression have been reported in a number of clinical studies [6]. It has been proposed that the 5-FC toxicity might be due to production of 5-FU by the intestinal flora [22]. Recently, by screening of the environmental metagenomic libraries, a novel isocytosine deaminase (ICD), termed Vcz, which specifically converts 5-fluoroisocytosine (5-FIC) into 5-fluorouracil (5-FU), has been identified [23]. We predict that the gut flora would not metabolize 5-FIC as extensively as it does in the case of 5-FC. Although it was shown that the CD of *E. coli* is able to catalyze the deamination of isocytosine, the kinetic parameters (k_cat_/K_m_) differ by the tenfold in favor of cytosine [24]. Moreover, to our knowledge, there are no known homologues of the ICD enzymes in the human cells. The new enzyme/prodrug pair, ICD/5-FIC, would alleviate the toxic side effects, which persist in the CD/5-FC system, and thus could be used as a novel enzyme-prodrug pair in the cancer therapy.

The aim of this study is to test the applicability of the ICD Vcz as a potential suicide gene therapy tool. We performed a series of cytotoxicity experiments in vitro experiments by using human originated cancer cell lines and, by employing MSC as the carrier of ICD/5-FIC, and, using the tumorized mice, tested the newly proposed gene therapy system in vivo.

## Methods

### Plasmid constructs

The DNA fragment, encoding human codon-optimized version of FLAG-tagged isocytosine deaminase Vcz (the sequence information is presented in the Additional file 1), which is flanked by the unique restriction sites BamHI and EcoRI, have been synthesized and subcloned into pUC57 (resulting to pUC57/Vcz) by GeneScript. The bicistronic vector pTO/Vcz-IG, encoding FLAG-Vcz and EGFP proteins, separated by the IRES element, was constructed in two steps. First, an intermediate vector pTO/Vcz was generated by subcloning the DNA fragment that encodes the FLAG-Vcz into vector pcDNA4/TO (Invitrogen) by using BamHI and EcoRI restriction sites. In the second step, the NotI – NotI fragment that contains IRES-EGFP of the lentiviral construct LeGO-iG2-RUNX3, which was a generous gift from Professor Yoshiaki Ito (Cancer Science Institute of Singapore, National University of Singapore, Singapore) was subcloned into pTO/Vcz plasmid linearized by NotI. The FLAG-Vcz-encoding letiviral vector pCSII/FVcz was generated by subcloning the blunted (with Klenow fragment) BamHI and NotI fragment from pUC57/Vcz into the lentiviral vector CSII-CMV-MCS-IRES2-Venus (kind gift from late Professor Lorenz Poellinger, Department of Cell and Molecular Biology, Karolinska Institute, Sweden), linearized by EcoRI and blunted with the Klenow fragment.

### Lentivirus production

Lentiviral vector stocks were produced by co-transfecting 2.5 μg of the FLAG-Vcz-encoding lentiviral vector pCSII/FVcz with 7.5 μg of packaging mix plasmids pLP1, pLP2 and pLP/VSVG (Invitrogen) into subconfluent monolayer cultures of 293FT cells on 10 cm Petri dish using jetPrime transfection reagent (Polypus). Supernatants were harvested after 72 h, clarified by low-speed centrifugation (1000 *g*, 10 min), filtered through 0.45-μm pore-size filter, aliquots were fast-frozen in liquid nitrogen, stored at − 80 °C. To determine vector titers, supernatant stock dilutions of 1:10, 1:50, 1:100 and 1:1000 were used to infect cultures of COS7 cells, and Venus-positive fluorescent cell colonies (transduction units (TU)) were counted after 48 h using a fluorescence microscope. The expression of FLAG-Vcz protein in transduced cells was verified by Western blot analysis using the FLAG tag-specific antibodies (described below).

### Cell culturing, transfection and transduction

The human glioblastoma cell line U87MG was obtained from the European Collection of Cell Cultures (ECACC, catalog No. 89081402), the GL261 murine glioblastoma cell line was a gift from Dr. Katalin Lumniczky (National Research Directorate for Radiobiology and Radiohygiene, Budapest, Hungary). The human embryonal kidney-derived cell lines 293H and 293FT were obtained, respectively, from Thermo Scientific (produced by Gibco, catalog No. 11631–017) and Invitrogen (catalog No. R70007). The human epithelial colorectal adenocarcinoma cell line Caco-2 was obtained from the American Type Culture Collection (ATCC, HTB­37). U87MG and GL261 cells were routinely propagated in Dulbecco’s Modified Eagle’s Medium (DMEM; Gibco), whereas 293H, 293FT and Caco-2 cells were grown in DMEM/Nutrient Mixture, F-12 Ham (Gibco) supplemented with 10% fetal bovine serum (FBS), 50 U/ml penicillin, and 50 g/ml streptomycin and were incubated at 37 °C in an atmosphere consisting of 95% air and 5% CO_2_ with 80% humidity in a humidified incubator. Mouse bone marrow mesenchymal stem cells (MSCs) were isolated from femur bones of 8 weeks of age C57/BL6 mice. Mice were sacrificed by cervical dislocation. The extracted bones were placed on a Petri dish containing phosphate buffered saline (PBS). Bone marrow was obtained by flushing with sterile PBS through one of the epiphyses, using a syringe needle (27-gauge). The bone marrow cells were collected in sterile PBS and washed three times by centrifugation for 6 min at 300 *g*. The centrifuged cells were seeded in T25 tissue culture flask and cultivated for up to 5 days in DMEM media supplemented with 10% FCS and high glucose (4.5 g/L). After 5 days, medium was changed and the floating cells were discarded, the attached cells were cultivated until 70% confluence and spread into the bigger flask for expansion procedures; growth medium was changed each 3 days.

Transient transfections were performed by introducing 0.5 μg of respective plasmid into cells grown in 22 cm^2^ Petri dishes by using Lipofectamine 2000 (Invitrogen) according to manufacturer’s recommendations. After transfection, cells were grown in DMEM for 48 h, and the cells were treated with 5-FIC, 5-FU or the DMSO as detailed in the figure legends. U87Vcz-IG cell line, which stably expressed Vcz and EGFP, was generated by transfecting U87MG cells with linearized (ScaI restriction) vector pTO/Vcz-IG and performing a multiple rounds of selection of cells that were resistant to zeocine (500 μg/ml). At the final stage of selection, the individual clones were isolated, propagated and examined for the expression of both Vcz and EGFP by Western blot analysis and fluorescence microscopy, as described below in this section.

The lentiviral transduction of MSCs was performed by incubating 6.4 × 10^6^ cells with Vcz lentivirus-containing cell supernatants (1.2 × 10^6^ TUs) in the presence of Polybrene at 10 μg/ml for 48 h. The medium was then replaced with fresh DMEM containing 10% FBS, and cells were grown for additional 72 h before injection into mice. The transduction efficiency was evaluated by the fluorescence microscopy.

The fluorescence emitted by cells upon their expression of EGFP (from either transiently or stably introduced bicistronic vector pTO/Vcz-IG) or Venus (upon lentiviral transduction) was monitored using Lumascope LS620 with the Lumaview 720/600-Series software (Etaluma Inc.), equipped with a FITC-filter set and the digital camera with the CMOS sensor.

### Whole-cell extract preparation and the Western blot analysis

Whole-cell extracts of transiently transfected U87MG and 293H cells, stable cells U87Vcz-IG, and 293FT cells transduced with the FLAG-Vcz letivirus were routinely prepared by resuspending the cell pellet in RIPA lysis buffer (50 mM Tris-HCl, pH 7.5, 150 mM NaCl, 1% Igepal CA-630, 0.5% sodium deoxycolate, 0.1% SDS) supplemented with a protease inhibitor cocktail (Sigma-Aldrich). Subsequently, the extracts were cleared by centrifugation for 30 min at 13.000 *g* at 4 °C. Eighty μg of the total extract protein were fractionated by 7.5% SDS-PAGE and transferred onto the nitrocellulose membranes. After the blocking overnight in 5% nonfat milk dissolved in phosphate-buffered saline (PBS), the immobilized proteins were incubated for 3 h at 25 °C with the primary rabbit polyclonal antibody against FLAG epitope tag (Thermo Scientific, catalog No. PA1-984B), dilution 1:1000) in blocking solution. After extensive washing in PBS-T buffer (PBS supplemented with 0.5% Tween-20), membranes were incubated with the horseradish peroxidase- (HRP-) conjugated anti-rabbit secondary antibody (Thermo Scientific, catalog No. 65–6120, dilution 1:2000) for 40 min. at 25 °C. Immunocomplexes were visualized using a chromogenic substrate 3,3′,5,5′-tetramethylbenzidine (TMB, Sigma-Aldrich) and documented by image scanner.

### MTT assay

The viability of cells grown and treated with chemicals in 96 well plates was examined by using the Vybrant MTT Cell Proliferation Assay Kit (Invitrogen, catalog No. V13154) according to manufacturer’s recommendations with the slight modification of the recommended protocol. Briefly, cells were washed with PBS and 80 μl of the MTT/cell medium mixture (0.5 μg/ml of the final MTT reagent concentration) was added to each well. After 3 h of incubation at 37 °C, this mixture was replaced by 100 μl DMSO. The intensity (absorbance) of the colored formazan product was measured at 550 and 620 nm by Multiskan GO Microplate Spectrophotometer (Thermo Fisher Scientific). Prior to performing the statistical analysis, the obtained data was processed in two steps: first the 620 nm measurements (background) was subtracted from individual 550 nm measurements, next, the data obtained from cell sample groups treated with either 5-FIC or 5-FU was normalized against the sample group treated with DMSO.

### Mouse glioblastoma model and cell/drug administration

Mouse glioblastoma model was tested by injecting 2 × 10^5^ GL261 cells into C57/BL6 mice subcutaneously into the abdomen area. After tumor inoculations, mice were followed for tumor growth once per 5 days. When visible tumors appeared (day 24), the experimental treatment with MSCs and prodrugs was initiated. Cells and drugs were injected intratumorally using BD 1 ml syringe with 27G needle. Cells were diluted in 0.9% NaCl solution and a volume of 100 μl was used per injection. (Pro) drugs (5-FIC and 5-FU) were diluted in DMSO, and a final volume of 50 μl was used for injection. When cells and drugs were co-injected, they were first mixed together prior to injection into the mouse. Treatment plan is displayed in the Table 1.

**Table 1:** Treatment plan of tumorized mice

	Treatment days after the visible tumor growth day 24 post-tumor inoculation						
Treatment groups (*N* = 10)	Day 0	Day 1	Day 2	Day 3	Day 5	Day 6	Day 7
Control group: 5-FU 20/50 mg per mouse	D		D		D		D
Control group: DMSO 50 μl per mouse	D		D		D		D
Control group: 10^6^ MSC-Vcz per mouse	C		C		C		C
Control group: 5-FIC 50 mg per mouse	D		D		D		D
Treatment group: 10^6^ MSC-Vcz per mouse + 5-FIC 20/50/100 mg per mouse	D + C	D	D + C	D	D + C	D	D + C

GL261-derived tumors evoked in C57/BL6 mice (10 mice in each treatment group) were treated with different injections, as indicated. MSC transduced with letivirus encoding FLAG-tagged Vcz designated as MSC-Vcz, treatments with chemicals only (DMSO, 5-FIC and 5-FU) are designated as *D*, treatments with MSC-Vcz cells only are designated as *C*, co-injections of MSC-Vcz and 5-FIC are designated as *D + C*.

### Statistical analysis

All grouped MTT analysis data are presented as mean ± standard deviation. Comparisons between groups were made by the unpaired t-test by using GraphPad Prism (version 5.0, GraphPad Software, Inc., San Diego, CA) and *p* < 0.05 was considered as statistically significant. Survival graphs were calculated using Kaplan-Meier method.

## Results

### Cytotoxicity test of 5-FIC on human glioblastoma U87MG and colorectal adenocarcinoma Caco-2 cell lines

**Fig. 1:**
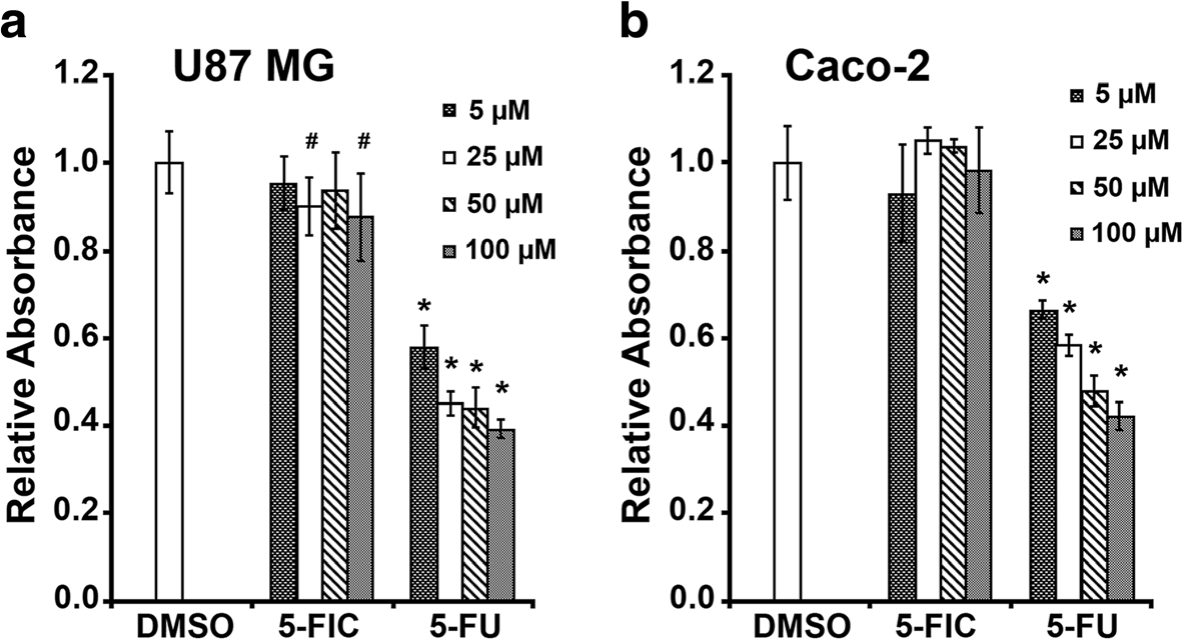
MTT assay of human glioblastoma U87MG and colorectal adenocarcinoma Caco-2 cell lines following treatment. **a** U87 MG and **b** Caco-2 cells were treated with different concentrations (indicated in the figure) of 5-FIC and 5-FU. DMSO was used as a vehicle control for both reagents. The *Relative Absorbance* values of 5-FIC and 5-FU treatment groups are shown as values obtained by measuring the intensity of the formazan at 550 nm, corrected for the background measured at 620 nm and normalized against the sample group treated with DMSO, the absorbance of which was considered as 1.0. Statistical significance indicated by *p*-values, where the hashtag symbol (#) designates *p* < 0.05, whereas the asterisk symbol (*) designates *p* < 0.0001 with respect to control (DMSO)

The MTT cell proliferation assay was carried out in order to test whether the pro-drug 5-FIC has any toxic effect on viability of human glioblastoma U87MG and colorectal adenocarcinoma Caco-2 cell lines. We used 5-FU (a drug) as a positive toxicity control, whereas DMSO was used as a vehicle control for both chemicals. According to the obtained results presented in Fig. 1, 5-FIC showed no significant effects on proliferation of either U87MG or Caco-2 cell lines even at the highest concentration tested (100 μM) (Fig. 1a and b, respectively). In contrast, 5-FU was cytotoxic to U87MG cells, the number of which decreased to 58% (at 5 μM) and further to 39% (at 100 μM), depending on a dose (Fig. 1a). Similarly, 5-FU was also cytotoxic to Caco-2 cells, the number of which decreased to 66% (at 5 μM) and further to 42% (at 100 μM) in a dose-dependent manner (Fig. 1b).

### Expression of Vcz in the mammalian cells

**Fig. 2:**
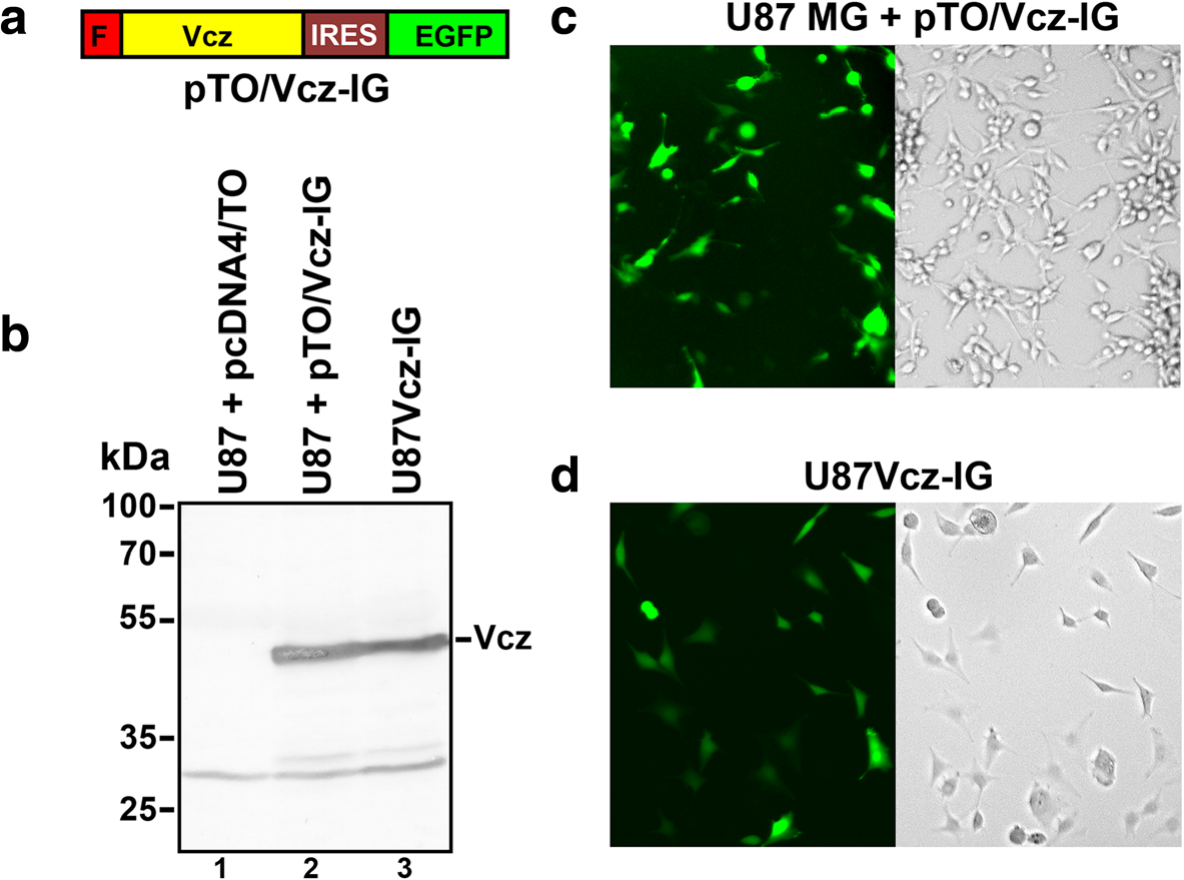
Expression of Vcz in the mammalian cells. **a** Schematic presentation of the pTO/Vcz-IG expression vector encoding, as indicated, the FLAG- (designated as *F*) tagged Vcz, IRES2 element (designated as IRES) and EGFP. **b** Expression of FLAG-tagged Vcz in U87MG cells transfected with the empty vector pcDNA4/TO (control, *lane 1*) and pTO/Vcz-IG (*lane 2*) and in the stable cell line U87Vcz-IG (*lane 3*) was detected by Western blot analysis, using FLAG-specific antibodies. The positions of the molecular mass standards (in kDa) and FLAG-Vcz protein (*Vcz*) are indicated. Expression of EGFP in **c** U87MG cells transfected with pTO/Vcz-IG and **d** in the stable cell line U87Vcz-IG were analyzed by fluorescence microscopy (left panels). Bright field views of the cells are presented in right panels

Identification and biochemical characterization of the bacterial isocytosine deaminase Vcz, which converts isocytosine into uracil and 5-FIC into 5-FU, was recently described in our recent publication [23]. On the basis of mammalian expression vector pcDNA4/TO, we constructed a bicistronic vector pTO/Vcz-IG, which encodes the FLAG-tagged human codon-optimized isocytosine deaminase Vcz, the enhanced green fluorescent protein (EGFP), and the internal ribosomal entry site (IRES2), which separates the two protein-coding sequences (Fig. 2a). We used this vector for the transient transfection experiments and for generation of the U87MG cell line, termed U87Vcz-IG, which stably expressed both Vcz and EGFP. The Western blot analysis was carried out on protein extracts prepared from U87MG cells transfected with pTO/Vcz-IG or empty vector pcDNA4/TO and from the stable cell line U87Vcz-IG. The FLAG-specific antibodies revealed a single 50.8 kDa band, which was present only in cells transfected with pTO/Vcz-IG and in the stable cell line U87Vcz-IG (Fig. 2b). Fluorescence microscopy imaging confirmed the expression of EGFP by U87MG cells transfected with pTO/Vcz-IG (Fig. 2c) and by stable cells U87Vcz-IG (Fig. 2d). These results confirmed that the bicistronic DNA construct was effective in driving the expression of FLAG-Vcz together with the fluorescent expression marker EGFP in cells of human origin.

**Fig. 3:**
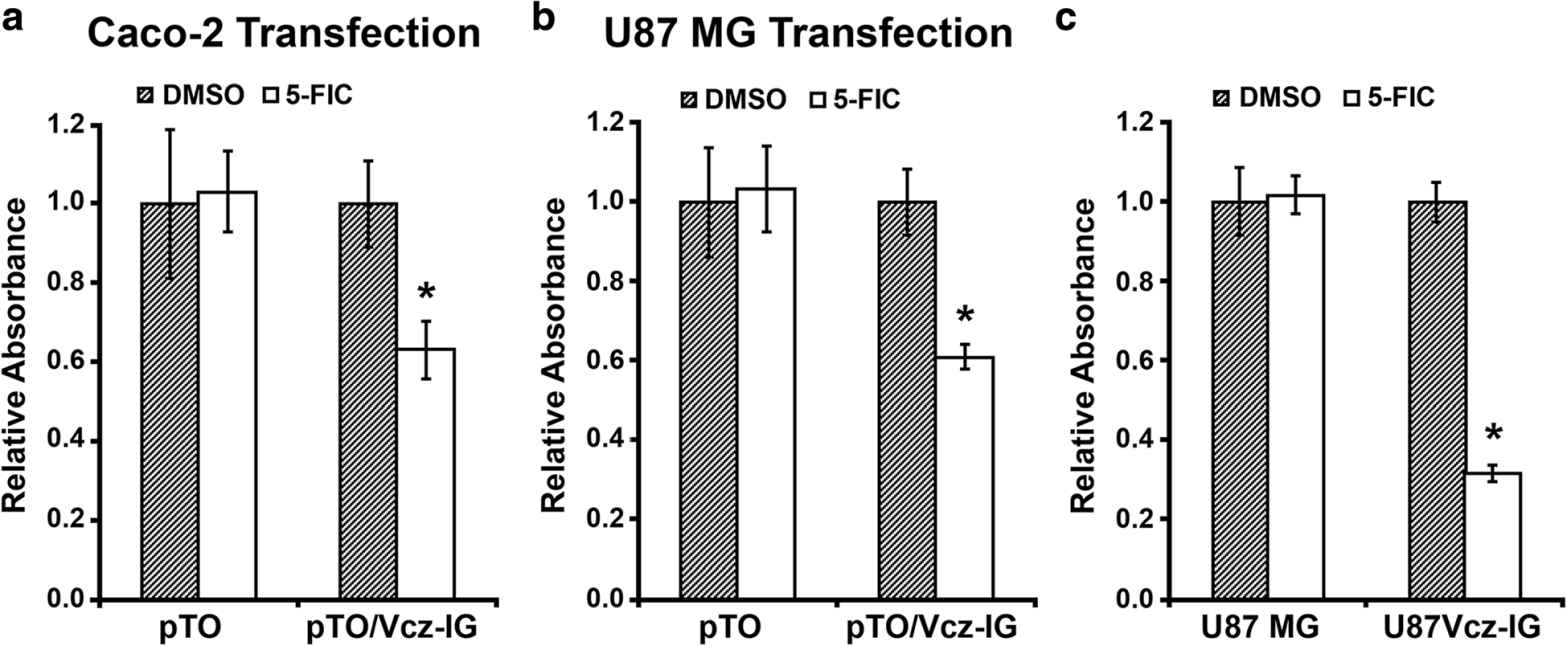
The cytotoxicity test of 5-FIC methabolic product excreted by Vcz expressing cells. **a** U87 MG and **b** Caco-2 cells were transiently transfected with Vcz-encoding vector pTO/Vcz-IG or control vector pcDNA4/TO (designated as *pTO*) and subsequently treated with 100 μM 5-FIC or DMSO for the additional 72 h. The calculations of *Relative Absorbance* values of treatment groups were carried out in the same way as for the Fig. 1. **c** U87 MG and Vcz stably-expressing cells U87Vcz-IG were treated the same way as transfected cells in a and b. Statistical significance indicated by *p*-values, where the asterisk symbol (*) designates *p* < 0.0001 with respect to control (DMSO)

As noted earlier, the isocytosine deaminase Vcz was originally synthesized in bacteria, where it was confirmed to possess deaminase activity on the substrate 5-FIC, which is converted by the enzyme into the cytotoxic product 5-FU. In the next two series of experiments, we wanted to verify whether Vcz protein retained its enzymatic activity while expressed in the mammalian cells. In the first series of experiments, U87MG and Caco-2 cells were transiently transfected with the FLAG-Vcz-encoding plasmid pTO/Vcz-IG or empty vector pcDNA4/TO (control) for 48 h followed by cell treatment with 100 μM 5-FIC or DMSO (vehicle control) for the additional 72 h. The viability of treated cells was evaluated using MTT cell proliferation assays. As presented in Fig. 3, the viability of both Caco-2 (Fig. 3a) and U87MG (Fig. 3b) cells transfected with pTO/Vcz-IG was reduced to approximately 60% in the presence of 5-FIC compared to cells transfected with the control vector or when cells were treated with the DMSO alone. Cationic lipids such as Lipofectamine used in this experiment are known to elicit an intrinsic cytotoxicity in different types of cells, including the cells studied in this work. We estimated that the toxicity of the Lipofectamine/DNA complexes on U87MG cells could reach up to 15% under our experimental conditions (data not shown). Therefore, in order to eliminate the extraneous toxic effects caused by transfection, we performed 5-FIC treatment of U87Vcz-IG cells, which stably expressed Vcz. As shown in Fig. 3c, we observed a marked reduction of number of U87Vcz-IG cells (down to approximately 30%) treated with 5-FIC, which was more pronounced when compared to the transiently transfected parental U87MG cells (Fig. 3b). The differences obtained by comparing transfected U87MG and stable U87Vcz-IG cell lines could be explained by the fact that we normally achieved up to 55% of the transfection efficiency on U87MG cells, whereas every single cell expressed Vcz protein in the stable line U87Vcz-IG. On the other hand, the fact that a significant fraction of U87Vcz-IG cells remained unaffected by 5-FIC treatment even though they expressed Vcz, suggested that a sub-population of cells within the U87MG cell line existed with some degree of resistance to 5-FU (which is a Vcz / 5-FIC product). It is yet to be determined whether the resistance to 5-FU can be overcome by the prolonged incubation of U87 MG cells with drug alone or in combination with another kind of treatment.

**Fig. 4:**
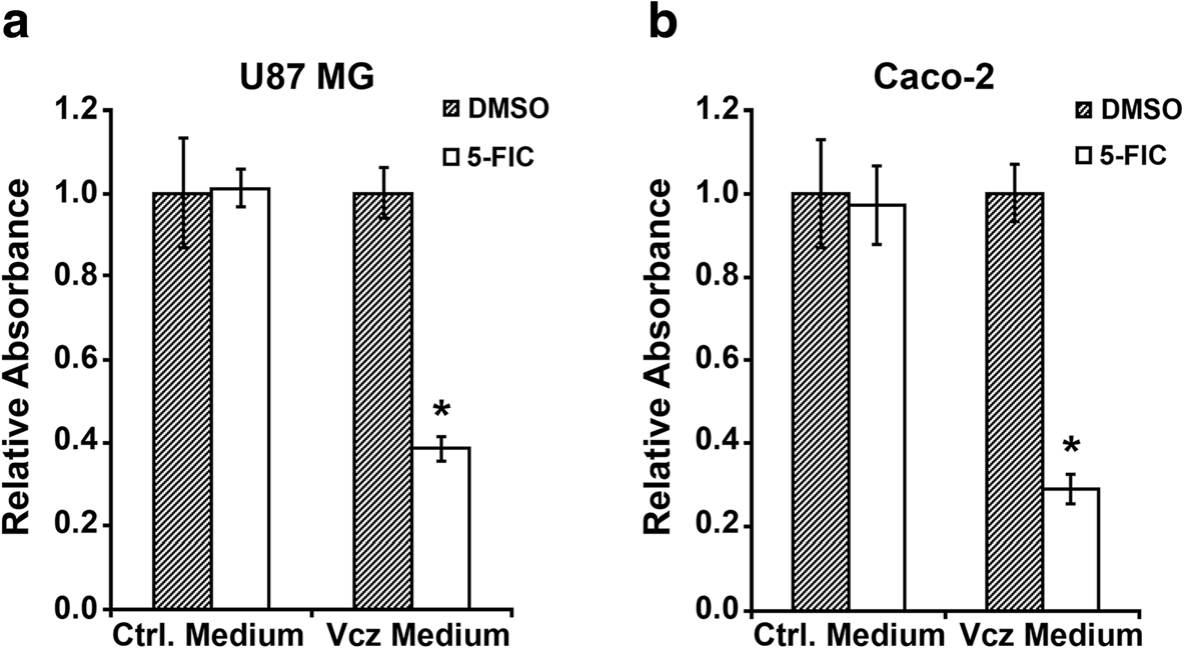
5-FIC cytotoxicity test of Vcz-expressing Caco-2 and U87MG cell lines. The cells were incubated with media collected from 293H cells, which were transfected with vectors pcDNA4/TO or pTO/Vcz-IG and treated with 100 μM 5-FIC or DMSO for 48 h. The growth media from treated control (designated as *Ctrl. Medium*) and Vcz-expressing cells (designated as *Vcz Medium*) was collected and applied onto **a** Caco-2 and **b** U87 MG cells. The calculations of *Relative Absorbance* values of treatment groups were carried out in the same way as for the Fig. 1. Statistical significance indicated by *p*-values, where the asterisk symbol (*) designates *p* < 0.0001 with respect to cell transfected with pcDNA4/TO and treated with DMSO

In the second series of experiments, we aimed to investigate whether the Vcz-mediated cytotoxic effect was only restricted to Vcz-expressing cells or it also manifested extracellularly, i.e. due to the emission of the newly produced 5-FU by the deaminase-expressing cells to the growth medium. For this purpose, 293H cells (chosen for this experiment because of their high transfectability) were transfected with the Vcz-encoding plasmid pTO/Vcz-IG or empty vector pcDNA4/TO and treated with 100 μM 5-FIC or DMSO for 48 h. Subsequently, the growth media was collected and applied onto U87MG and Caco-2 cells, followed by incubation for 72 h. The viability of U87MG and Caco-2 cells was evaluated using the MTT assay. As shown in Fig. 4, the number of both 87MG (Fig. 4a) and Caco-2 (Fig. 4b) cells was markedly decreased upon incubation of cells with the medium collected from Vcz-expressing 293H cells that were treated with 5-FIC. Taken together, these results are in line with our expectation that the ectopically expressed Vcz converts the non-toxic producing 5-FIC into 5-FU, which is subsequently released into the extracellular milieu.

### Effect of Vcz-expressing MSCs on in vivo tumor growth

**Fig. 5:**
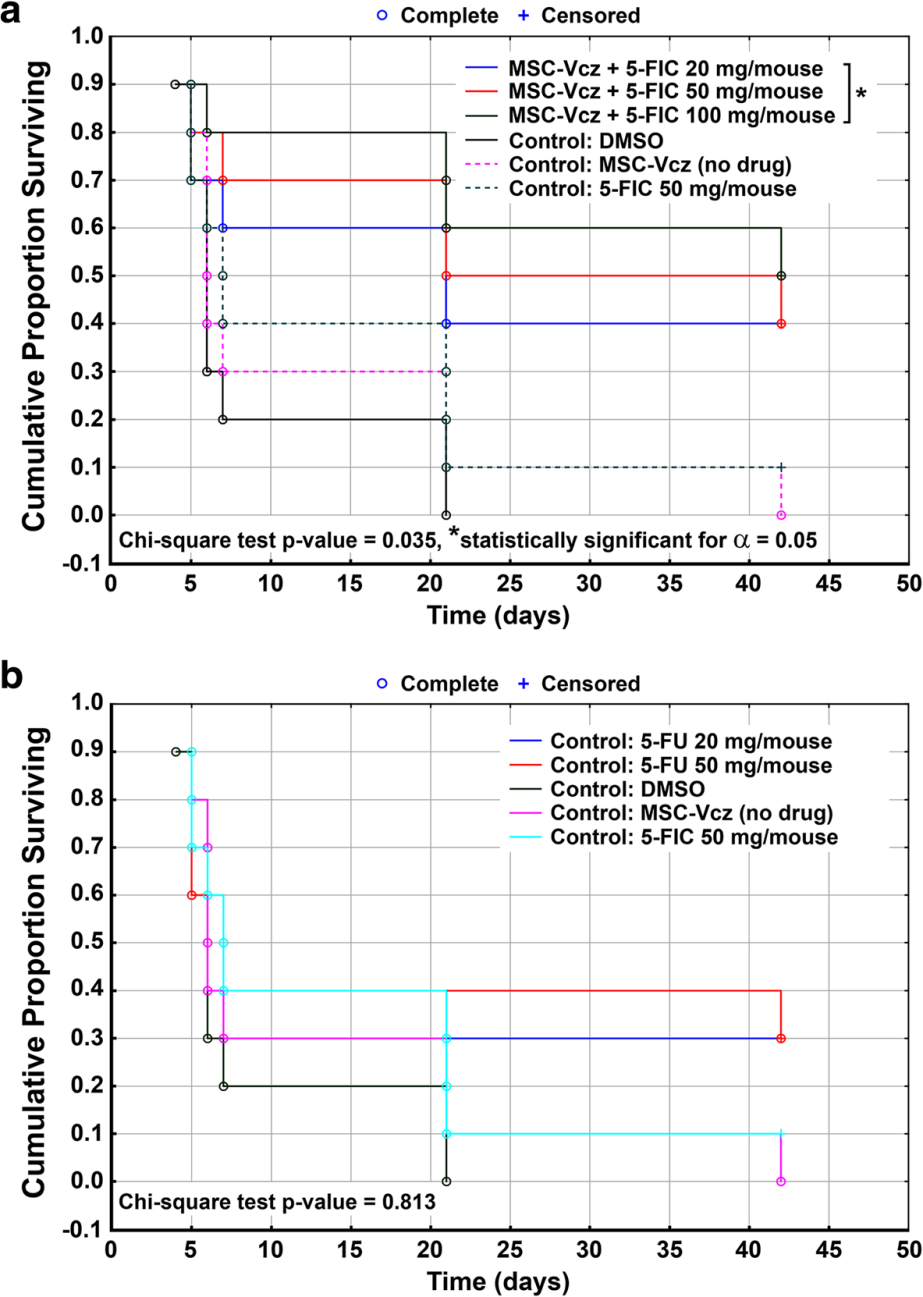
Kaplan-Meier survival analysis of tumorized mice following different treatments. **a** Subcutaneous GL261 tumors evoked in C57/BL6 mice were treated with co-injections of MSC transduced with Vcz-encoding lentivirus (MSC-Vcz) and different concentrations of 5-FIC, as indicated. In control mice, intratumoral injection of DMSO, MSC-Vcz cells alone (without 5-FIC), 5-FIC alone was used. These control treatment results are shown separately in panel **b** including additional intratumoral treatments with different concentrations of 5-FU, as indicated. The survival time (number of days) of mice upon different intratumoral treatments and the chi-square test *p*-values estimated for this experiment, are indicated. The asterisk symbol (*) marks the statistical significance between MSC-Vcz + 5-FIC and control treatment groups, determined by the *p*-value = 0.035 of the Chi-square test

**Fig. 6:**
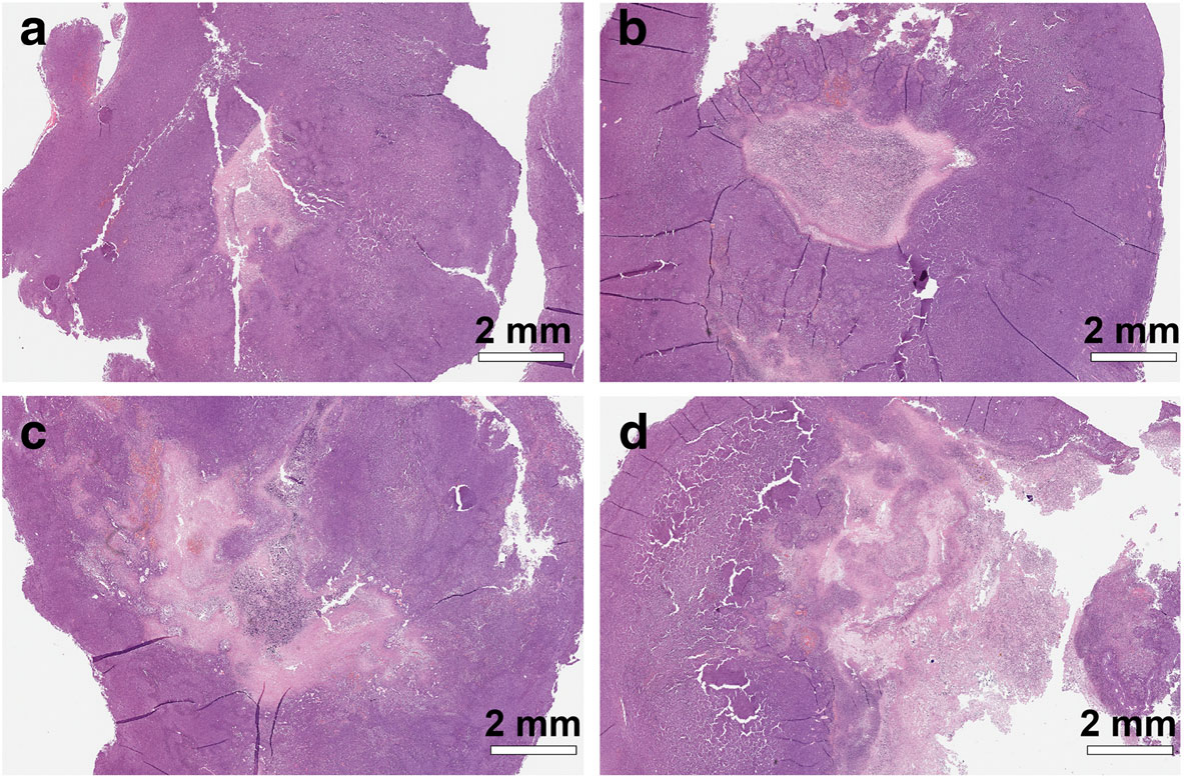
Histological analysis of tumor necrosis after different treatments. Hematoxylin and eosin staining of GL261 tumors treated with **a** DMSO, **b** 5-FIC (50 mg/mouse), **c** 5-FU (50 mg/mouse), and **d** MSC-Vcz and 5-FIC (50 mg/mouse) are shown. Bright pink areas reveal tumor necrosis progression. The scale bar of 2 mm is shown

In order to assess the anticancer potential of the new drug (5-FIC)/ enzyme (deaminase Vcz) pair in an in vivo system, we decided to use the murine brain tumor model GL261. We used murine bone marrow-derived MSCs as a carrier of Vcz, which was introduced into these cells via the lentiviral transduction. Histological analysis revealed infiltration of glioblastoma tumor into the site of tumor cell inoculation occurring 24 days after the GL261 injection into C57/BL6 mice (data not shown). Vcz lentivirus-transduced MSC (MSC-Vcz) with or without pro-drug 5-FIC as well as drug 5-FU at different concentrations and the vehicle DMSO were injected intratumorally according to the treatment scheme presented in the Table 1 (please see section “Methods”). The combined result of treatments is presented as Kaplan-Meier survival curves in Fig. 5a. The Fig. 5b shows all control treatments including MSC-Vcz, 5-FIC, 5-FU and DMSO. The obtained results indicate that the 5-FIC/Vcz system is 10 to 20% more efficient than the drug 5-FU alone when comparing the mice survival in these groups. The higher 5-FIC efficacy over 5-FU was more apparent in the survival analysis when we compared three MSC-Vcz treatment groups made of pooled treatments: 1) in one group we combined treatments with all tested 5-FIC concentrations (20, 50 and 100 mg/mouse), 2) the second group contained treatments with all tested 5-FU concentrations (20 and 50 mg/mouse) and 3) the third treatment group was composed of MSC-Vcz controls (see the Additional file 2). The comparison of 5-FIC/Vcz groups and the control groups revealed that our new anticancer technology supported more than 50% of mice survival in the day 40 after the treatment (day 64 after the tumor inoculation). Histological staining of GL261 tumors after different treatments revealed that, when compared to control treatments with DMSO (Fig. 6a) and 5-FIC (Fig. 6b), the size of the necrotic area in tumors treated with drug 5-FU was increased (Fig. 6c) and it was enlarged even further in tumors treated with co-injection of MSC-Vcz and 5-FIC (Fig. 6d).

## Discussion

In our recent study, the metagenomic libraries and an *E. coli* uracil auxotroph-based selection strategy has been used to search for putative ICDs [23]. Since the expected enzymatic activity of the ICD is the conversion of isocytosine into uracil, we used an *E. coli* strain lacking several *pyr* genes that are responsible for the de novo biosynthesis of pyrimidines. This strain is unable to grow in the defined synthetic medium without a source of uracil, therefore, it was used as a host strain for screening of the metagenomic libraries – a pool of plasmid vectors containing random genomic DNA fragments from soil samples. The ICD discovered in our screens – was termed Vcz – was shown to catalyze the conversion of isocytosine into uracil as well as 5-FIC into 5-FU [23]. Importantly, cytosine has been demonstrated not to be a substrate for Vcz, arguing for the specificity of this enzyme towards the isomer of cytosine – isocytosine.

In this study, the majority of in vitro experiments were conducted on glioblastoma cell line U87MG, whereas the murine glioblastoma tumor model GL261 was used for in vivo experiments. 5-FU, as a product of the ICD Vcz/5-FIC activity, has been tested previously in a considerable number of glioma studies using mice and rat models, which subsequently lead to the initial clinical trials (phase I) showing promising results and prospects towards phase II trials [25, 26] (for reviews, see [27, 28]). The pharmacological effects of 5-FIC on human organism are yet to be explored, however, there is a reason to believe that the gut flora would not metabolize 5-FIC to the same extent as it has been reported for 5-FC [22]. Although it was shown that the CD from *E. coli* is able to catalyze the deamination of isocytosine, the kinetic parameters (k_cat_/K_m_) differ by the tenfold in favor of cytosine [24]. In addition, to our knowledge there are no known homologues of the isocytosine deaminase in the human cells. In this respect, the ICD/5-FIC system has a potential to rival with the well-established CD/5-FC system and thus is considered to have a great potential to be used a suicide gene therapy tool for cancer treatment. In this study, we showed that human codon-optimized Vcz gene expressed well in the mammalian cells, it was a stable protein (since we observed only a low amount of the degradation products compared to the major band of Vcz protein in Western blots (Fig. 2b)) and it was functional in producing cytotoxic product, which was excreted to induce the bystander’s effect (Fig. 4).

One of the main challenges that gene therapy faces is the delivery of the therapeutic agent to the specific target site. Delivery of the enzyme/prodrug system to the tumor site by MSC may be one possibility [19, 20]. MSC migrate to the site of injury where they promote the regenerative processes by structural repair of wounds via cellular differentiation, immune-modulation, production of growth factors that drive neovascularization and re-epithelialization, and mobilization of resident stem cell niche [29, 30]. The tumor stroma contains a number of elements (such as blood vessels, inflammatory cells, connective tissue together with matrix elements), which are likened to wound healing and thus provide appropriate signals that attract MSC [31]. A plethora of therapies were designed on the basis of tumor tropism, in which MSC are being used as a vehicle for delivery of therapeutic agents to the tumor site. As an example, a study published by Kucerova and colleagues demonstrated that human adipose tissue-derived MSC, which express the fused yeast cytosine deaminase::uracil phosphoribosyltransferase (yCD::UPRT), augmented the 5-FC-mediated bystander effect and selective cytotoxicity on human colon cancer cells HT-29 in vitro and significantly inhibited HT-29-induced tumors in mice [32]. In similar studies reported by the same laboratory, yCD::UPRT-expressing MSC together with 5-FC proved to be therapeutically efficient in treatment of melanoma A375 xenografts [33] as well as the intracerebral rat C6 glioblastoma [34, 35].

In this study, we used murine bone marrow-derived MSC that were transduced with Vcz-encoding lentivirus (Vcz-MSC) for the injection into the mice tumorized with GL261cells. The co-injection of Vcz-MSC and FIC supported more than 50% of survival of mice compared to control injections (FIC or Vcz-MSC alone). Despite the obtained encouraging results, this particular experiment we considered as a “pilot” since there is a plenty of room remaining for the improvement of the experimental conditions that have been used so far. One of the major drawbacks of the described experiment was that only up to 10% of MSC transduction efficiency was achieved (see the Additional file 3). This problem could be solved by using higher titers of Vcz-encoding lentivirus or by generating stable Vzc-expressing immortalized MSC. The latter approach seems to be a solution for the common problem faced by MSC-based therapies since a rapid death of the prodrug-converting cells represents a major limitation. The immortalization of MSC can be achieved by introduction of the telomerase reverse transcriptase [36, 37] or simian virus 40 large antigen genes [38]. Previous studies have demonstrated that the phenotype of human MSC including their differentiation potential is maintained after immortalization [39, 40].

## Conclusions

In summary, during the proof-of-concept experiments, we demonstrated that the novel ICD Vcz system is a functionally active enzyme in human-derived cells, which, in combination with the specific substrate 5-FIC, has a potent cytotoxic effects on the surrounding cells. The novel ICD Vcz together with the non-toxic 5-FIC can be viewed as a new potential enzyme / prodrug system for suicide gene therapy, the efficacy of which will be determined by the development of the tumor-targeted enzyme delivery system. The use of MSC as a carrier of the Vcz / 5-FIC system was proven successful in reducing the aggressiveness of the tumor in mouse glioblastoma model.

## Additional files

Additional file 1: DNA sequence encoding FLAG-Vcz protein. Underined is the FLAG-encoding sequence. Respective protein synthesis start and stop codons are marked in bold. (DOCX 18 kb)

Additional file 2: Kaplan-Meier survival analysis of mice groups made of pooled MSC-Vcz treatments: 1) in one group we combined treatments with all tested 5-FIC concentrations (20, 50 and 100 mg/mouse), 2) the second group contained treatments with all tested 5-FU concentrations (20 and 50 mg/mouse) and 3) the third treatment group was composed of MSC-Vcz controls. The asterisk symbol (*) marks the statistical significance between MSC-Vcz + 5-FIC and control treatment groups, determined by the *p*-value = 0.01 of the Chi-square test. The Log rank value for 5-FIC vs control groups was determined as *p* < 0.001 (significant), whereas the Log rank value for 5-FU vs control groups was determined as *p* = 0.098 (not significant). (PDF 296 kb)

Additional file 3: MSC transduction with the letivirus encoding Vcz and EGFP. **a** Fluorescence image and (**b**) the bright field view of MSC cells transduced with letivirus. The scale bar of 100 μm is shown. (PDF 145 kb)

## Abbreviations

5-FC: 5-fluorocytosine
5-FIC: 5-fluoroisocytosine
5-FU: 5-fluorouracil
CD: Cytosine deaminase
DMSO: Dimethyl sulfoxide
ICD: Isocytosine deaminase
MSC: Mesenchymal stem cells
