# Supplementary material for: Isocytosine deaminase Vcz as a novel tool for the prodrug cancer therapy
Source: BMC Cancer. 2019 Mar 4;19:197. doi: 10.1186/s12885-019-5409-7 (PMC6399854; doi:10.1186/s12885-019-5409-7)
Supplement: Supplementary file 1 — DNA sequence encoding FLAG-Vcz protein. Underined is the FLAG-encoding sequence. Respective protein synthesis start and stop codons are marked in bold. (DOCX 18 kb) [file 12885_2019_5409_MOESM1_ESM.docx]

**DNA sequence encoding FLAG-Vcz protein**

**ATG**GACTACAAAGACGACGACGACAAAGGAGAATTCGATAAACGCACACTGCTGTTCAAAAACGCCGAGCTGCTGGTCACAATGGACGACGAGAGGAGAGAGATCAGGGGAGGATGCCTGCTGGTGGAGGGCAACAGAATCGTGGCAGTGGGAGGCGATGAGCTGTGCGCAGCACCTGCAGACGAGGAGATCGATCTGAGGGGCCACATCGTGATCCCAGGCCTGATCAATACCCACCACCACATGTTCCAGAGCCTGACAAGAGTGATCCCCGACGCCCAGGATGGCGAGCTGTTTGACTGGCTGAACAATCTGTACCCTATCTGGGCCGGCCTGACCCCAGAGATGATCAGGATCAGCACCCAGACAGCCATGGCCGAGCTGATGCTGTCCGGCTGCACCACAAGCTCCGACCACCTGTACGTGTATCCTAACGGCTGTAGACTGGACGATTCCATCGATGGAGCAAGGGAGATCGGAATGAGGTTCCACGCATGCAGGGGCTCTATGAGCGTGGGCCGGTCTAAGGGAGGACTGCCACCTGATGAGCTGGTGGAGAATGAGCAGGCCATCCTGGAGGACTCCCTGCGCCTGATCCACTCTTACCACGATGCCCAGAGATATTCTATGCTGAGGATCGCCCTGGCACCATGTAGCCCCTTTTCCGTGTCTCGGGAGCTGATGGTGAAGACCGCACAGATGGCAAGGGAGCAGGGCGTGTCCCTGCACACACACCTGGCCGAGAACGACTCTGATGTGAGCTACTCCCAGACCCACTTCGGCATGACACCAGCCCAGTATGCAGAGGACCTGGGATGGGTGGGCTCTGACGTGTGGCACGCCCACTGCGTGAAGCTGGACAGAGCAGGAATCAGCCTGTTTGCAAGGACCGGAACAGGAGTGGCACACTGCCCATGTAGCAATATGAGGCTGGCATCCGGAATCGCACCAATCAGGGCAATGCTGGATGAGGGCGTGAGCGTGGGACTGGGAGTGGACGGCTCTGCCAGCAACGATGCCGGCAATATGATCGCAGAGACCAGGCAGGCAATGCTGCTGCAGAGGGTGGGATTCGGACCAGACGCAATGAACGCAAGGCAGGCCCTGGAGATCGCAACAAGGGGAGGAGCCAAGGTGCTGAATAGAGACGATATCGGATATCTGGCAACCGGAATGGCAGCAGACTTCGTGGCCTTTGATCTGAACACACTGAATCTGGCAGGAGCAAAGCACGACCCACTGGCCGCCCTGGTGTTCTGTACCCCTGGCAACGTGGCCTTTTCCGTGATCAATGGACAGGTGGTCATCAGGGAGGGCGTGCTGCAGACAATCGATCTGCCTTCCGTGGTGCAGCAGCATAATCGCCTGGCATGTCTGCTGGTCAATCGGCATCGACTG**TAA**
