# Supplementary figures and images for: Isocytosine deaminase Vcz as a novel tool for the prodrug cancer therapy
Source: BMC Cancer. 2019 Mar 4;19:197. doi: 10.1186/s12885-019-5409-7 (PMC6399854; doi:10.1186/s12885-019-5409-7)

# Cumulative Proportion Surviving (Kaplan-Meier)

○ Complete + Censored

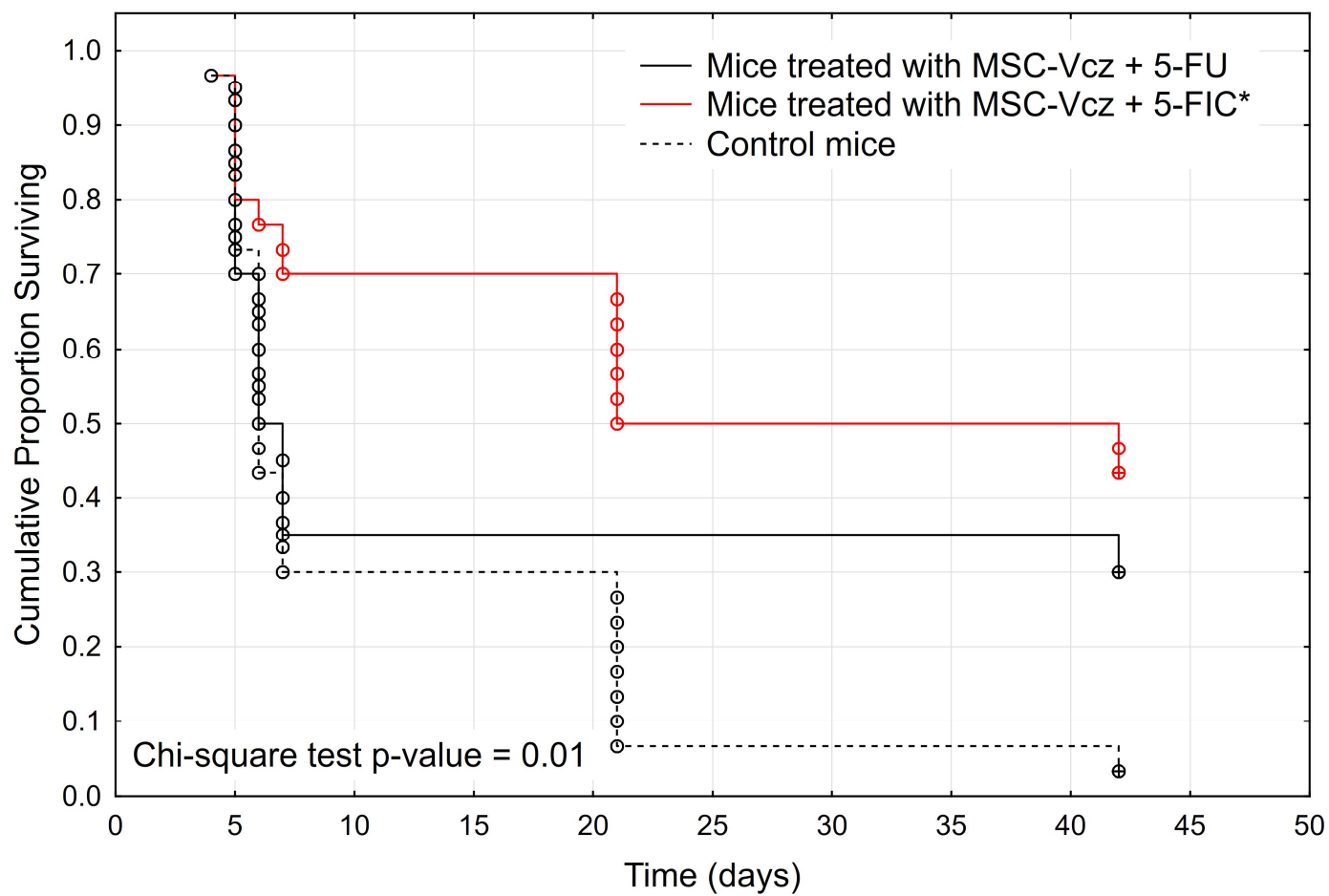

Supplement: Supplementary file 2 — Kaplan-Meier survival analysis of mice groups made of pooled MSC-Vcz treatments: 1) in one group we combined treatments with all tested 5-FIC concentrations (20, 50 and 100 mg/mouse), 2) the second group contained treatments with all tested 5-FU concentrations (20 and 50 mg/mouse) and 3) the third treatment group was composed of MSC-Vcz controls. The asterisk symbol (*) marks the statistical significance between MSC-Vcz + 5-FIC and control treatment groups, determined by the p-value = 0.01 of the Chi-square test. The Log rank value for 5-FIC vs control groups was determined as p < 0.001 (significant), whereas the Log rank value for 5-FU vs control groups was determined as p = 0.098 (not significant). (PDF 296 kb) [file 12885_2019_5409_MOESM2_ESM.pdf]

## MSC transduction with the letivirus encoding Vcz and EGFP

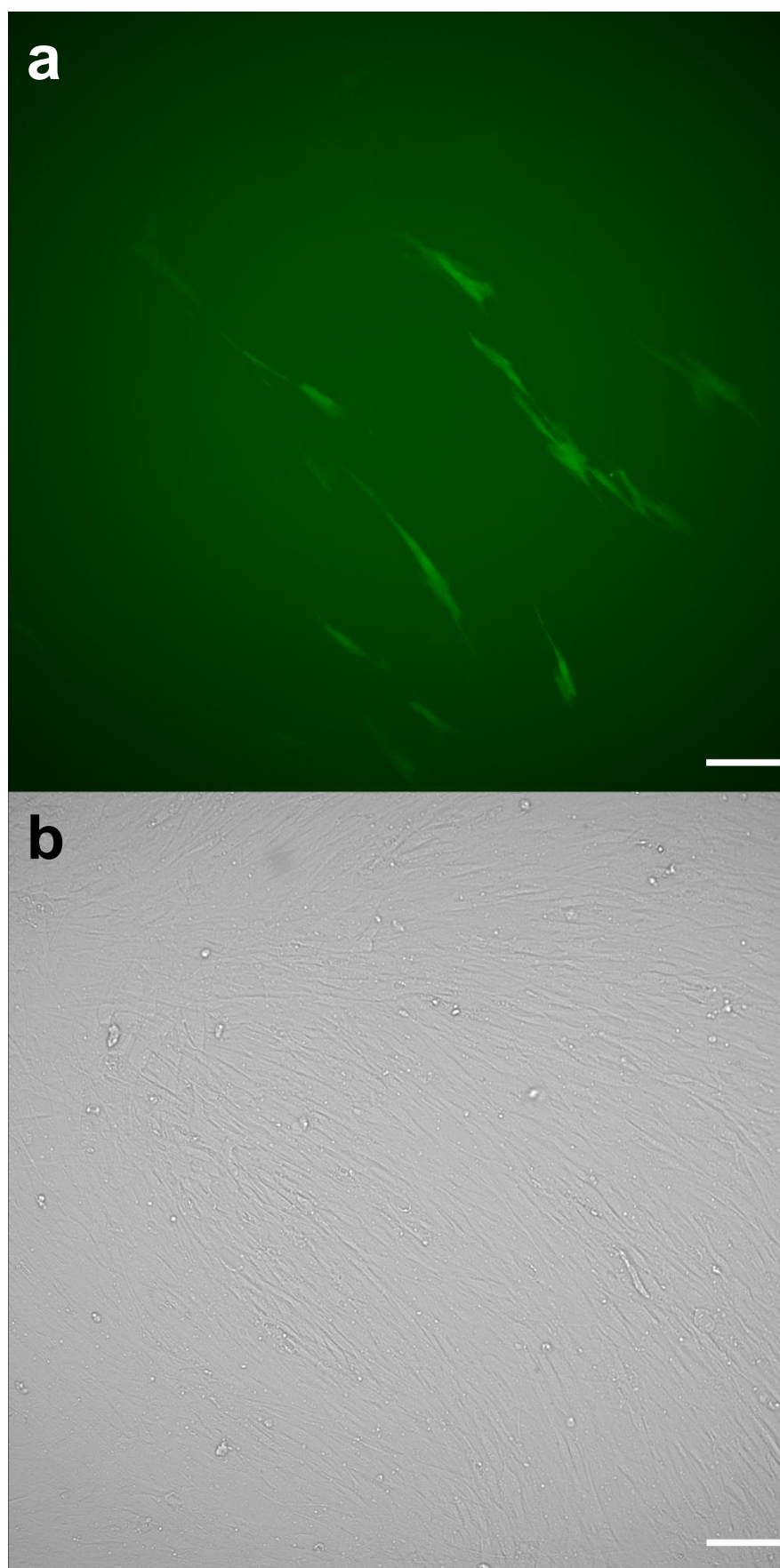

Supplement: Supplementary file 3 — MSC transduction with the letivirus encoding Vcz and EGFP. a Fluorescence image and (b) the bright field view of MSC cells transduced with letivirus. The scale bar of 100 μm is shown. (PDF 145 kb) [file 12885_2019_5409_MOESM3_ESM.pdf]
